# Association of the android to gynoid fat ratio with nonalcoholic fatty liver disease: a cross-sectional study

**DOI:** 10.3389/fnut.2023.1162079

**Published:** 2023-05-15

**Authors:** Ling Yang, Hangkai Huang, Zhening Liu, Jiaqi Ruan, Chengfu Xu

**Affiliations:** ^1^Department of Gastroenterology, The First Affiliated Hospital, Zhejiang University School of Medicine, Hangzhou, China; ^2^Zhejiang Provincial Clinical Research Center for Digestive Diseases, Hangzhou, China

**Keywords:** nonalcoholic fatty liver disease, dual-energy X-ray absorptiometry, android fat, gynoid fat, risk

## Abstract

**Background:**

Nonalcoholic fatty liver disease (NAFLD) is becoming a severe global public health problem, and can developed into fibrotic nonalcoholic steatohepatitis (NASH), but its risk factors have not been fully identified. The objective of this study was to investigate the association between the android-to-gynoid fat ratio (A/G ratio) and the prevalence of NAFLD.

**Methods:**

This cross-sectional study is based on the 2003–2006 and 2011–2018 cycles of the National Health and Nutrition Examination Survey and included 10,989 participants. Participants aged 20 and older without viral hepatitis or significant alcohol consumption were included. Dual-energy X-ray absorptiometry was used to assess body composition. NAFLD was diagnosed using the United States fatty liver index (US FLI). Multivariable logistic regression models were used to evaluate the association between the A/G ratio and NAFLD.

**Results:**

The prevalence of NAFLD was 32.15% among the study population. Android percent fat and the A/G ratio were significantly higher in patients with NAFLD than in those without NAFLD [41.68% (0.25) vs. 32.80% (0.27), *p* < 0.001; 1.14 ± 0.01 vs. 0.94 ± 0.00, *p* < 0.001, respectively]. Logistic regression analysis showed that android percent fat was positively correlated to NAFLD (OR: 1.15, 95% CI: 1.11–1.18), while gynoid percent fat was negatively correlated to NAFLD (OR: 0.92, 95% CI: 0.90–0.94), and the A/G ratio was significantly associated with the prevalence of NAFLD (OR: 1.59, 95% CI: 1.38–1.82) and fibrotic NASH (OR: 2.01, 95% CI: 1.71–2.38). We also found that females had a notably diminished A/G ratio compared with males (0.91 vs. 1.12, *p* < 0.001). In addition, the female population proportion was negatively correlated with the A/G ratio, which may partly explain the lower prevalence of NAFLD in females. What is more, the OR value of the A/G ratio in the female subgroup was much higher than that in the male subgroup in all adjusted models.

**Conclusion:**

A/G ratio is significantly associated with NAFLD and fibrotic NASH. Women have a lower A/G ratio than men, which may explain the sex difference in NAFLD prevalence. Furthermore, with a higher A/G ratio, the association between females and NAFLD are greatly elevated.

## Highlights

- A/G ratio is significantly related to NAFLD and fibrotic NASH, which provides a novel and vital indicator of NAFLD for individuals in health screening in the future.
- An increased A/G ratio reverses the previously observed weaker association between NAFLD and women. This indicated that the A/G ratio played a key role in the development of NAFLD in females. Investigating the mechanisms behind the link between the A/G ratio and NAFLD may provide a novel approach for the prevention and treatment of the disease.

## Introduction

Nonalcoholic fatty liver disease (NAFLD) is a progressive liver condition that can manifest from simple steatosis to steatohepatitis, fibrosis, and even hepatocellular cancer (1, 2). In recent decades, the prevalence of NAFLD has risen alarmingly from 25% in 2018 to 32.4% in 2022, making it the leading cause of chronic liver diseases worldwide (3, 4). It has significantly increased the morbidity and mortality rates linked to advanced liver disease, diabetes mellitus, and cardiovascular events (5, 6).

As NAFLD is becoming a severe worldwide public health problem, efforts to identify risk factors for NAFLD have become a research priority. Although there have been reports of several risk factors for NAFLD, such as genetic predisposition, diabetes, metabolic syndrome, and limited medical access, the risk factors for NAFLD have not been fully clarified (7–9). Obesity is the most important risk factor for NAFLD (10–15) and is commonly assessed using weight, body mass index or waist circumference. However, these indicators were questioned as not being the best measures (16). Dual-energy X-ray absorptiometry (DEXA) is one of the most precise direct measurements of adipose tissue distribution and quantity and may provide more basic evidence for the association between obesity and NAFLD.

The latest research showed that women had a significantly lower prevalence of NAFLD than men (3). Moreover, the pathogenesis of the sex-related epidemic of NAFLD remains unknown. Previous studies have revealed notable sex differences in fat distribution. The android fat pattern, an “apple-shape” in which fat accumulates mostly in the abdomen, is reported to be more common in males and is associated with an increased risk of type 2 diabetes and atherosclerosis, while the gynoid fat pattern, a “pear-shape” in which fat accumulates mostly around the hips and femur, known as a “female” fat distribution, carries much less risk (17, 18). These two fat depots might interact with NAFLD, but no large cross-sectional study has investigated this interaction before. Whether the two sex-related fat depots are correlated with NAFLD needs further exploration.

This study aimed to examine whether there is an independent association between android and gynoid fat and the presence of NAFLD. We also appraised the sex-specific association of android and gynoid fat with NAFLD prevalence.

## Methods

### Study participants

The National Health and Nutrition Examination Survey (NHANES) is a national and cross-sectional health and nutrition examination and survey that is organized regularly every 2 years by the National Center for Health Statistics (NCHS), addressing diverse U.S. population groupings and health issues. We included NHANES 2003–2006 and 2011–2018 cycles involving 59,626 sampled participants in our study due to the availability of DXA data. We studied a subgroup of 13,287 people aged 20 and older with fasting laboratory measures. Participants were excluded if they had a positive serum hepatitis B surface antigen or hepatitis C antibody or if their hepatitis serological information was missing (*n* = 667). Additionally, we excluded those who drank heavily (one or more drinks per day for females and two or more drinks per day for males) and those who lacked information on alcohol consumption (*n* = 1,631). Finally, 10,989 individuals were included in this study (Supplementary Figure S1).

### Definitions of NAFLD and fibrotic NASH

The Fatty Liver Index (FLI) is a simple and accurate predictor of hepatic steatosis in the general population (19), which had already been validated by magnetic resonance spectroscopy (20, 21). As the participants in this study were from the United States, NAFLD was determined using a modified version of the FLI—the United States Fatty Liver Index (US FLI)—developed by Ruhl et al. (22), with a cutoff value of 30. The US FLI set up on the NHANES 1988–1994 data for predicting fatty liver in the multiethnic U.S. population. It was estimated using the following variables: ethnicity, age, gamma-glutamyl transferase, waist circumference, fasting insulin, and fasting glucose. The formula was calculated as follows: US FLI = (e^−0.8073 * non-Hispanic black + 0.3458 * Mexican American + 0.0093 * age + 0.6151 * log^_e_^(GGT) + 0.0249 * waist circumference + 1.1792 * log^_e_^(insulin) + 0.8242 * log^_e_^(glucose) − 14.7812^)/(1 + e^−0.8073 * non-Hispanic black + 0.3458 * Mexican American + 0.0093 * age + 0.6151 * log^_e_^(GGT) + 0.0249 * waist circumference + 1.1792 * log^_e_^(insulin) + 0.8242 * log^_e_^(glucose) − 14.7812^) * 100.

Fibrotic nonalcoholic steatohepatitis (NASH) was identified using the Fibrotic NASH Index (FNI), developed by Tavaglione et al. (23), with a cutoff of 0.33. The FNI incorporates the following variables: aspartate aminotransferase (AST), high-density lipoprotein cholesterol (HDL), and hemoglobin A1c (HbA1c). The formula was calculated as follows: FNI = (e^(−10.33 + 2.54 * log^_e_^(AST) + 3.86 * log^_e_^(HbA1c) − 1.66* log^_e_^(HDL))^)/(1 + e^(−10.33 + 2.54 * log^_e_^(AST) + 3.86 * log^_e_^(HbA1c) − 1.66 * log^_e_^(HDL))^).

### Dual-energy X-ray absorptiometry

Dual-energy X-ray absorptiometry (DXA) was applied to estimate body adipose amounts. Android is defined as having fat distribution around the midsection or waist (belly button). Gynoid refers to the area of the hips that is located at the tops of the thighs. The NHANES DXA android/gynoid measurement data set was used to extract android and gynoid body fat measurements and the android/gynoid ratio (A/G ratio). The lower trunk region enclosed by the pelvic line and the horizontal cut line of the pelvis was referred to as the “android area.” Below the pelvic line, the height of the android region was 1.5 times as tall as the upper gynoid line, and the height of the android region was 2 times as tall as the distance between the two gynoid lines. Hologic software automatically added the lines indicated above (24–29).

### Clinical parameters and biochemical tests

Baseline characteristics such as age, sex, ethnicity/race, educational degree, family income-to-poverty ratio (PIR), and marital status were extracted from the demographic data sets. Anthropometric measures, including height, weight, body mass index (BMI), waist circumference, and blood pressure, were extracted from examination data. Laboratory data such as triglycerides, total cholesterol, high-density lipoprotein (HDL) cholesterol, low-density lipoprotein (LDL) cholesterol, alanine aminotransferase (ALT), aspartate aminotransferase (AST), free fatty acids, fasting blood glucose, insulin, glycohemoglobin, and uric acid were collected. Insulin resistance was evaluated by the homeostasis model assessment of insulin resistance (HOMA-IR): HOMA-IR = [fasting insulin (U/mL) × fasting serum glucose (mmol/L)/22.5] (30).

### Statistical analysis

As NHANES is survey data with a complex, multistage sampling design, we created 6-year fasting subsample Mec weights (WTSAF2YR*1/6) to combine survey cycles (2003–2006 and 2011–2018). Masked variance pseudostrata and variance pseudo-PSU were also included to define the survey design. The prevalence and prevalence ratio were calculated as reported before (31, 32). For continuous variables on demographic characteristics, anthropometric measurements, and laboratory information, data are shown as the means and standard errors (SEs), and for categorical variables, data are displayed as numbers (percentages).

The differences between groups were compared with Student’s *t*-test or *F* test for continuous variables and the Rao-Scott *χ*^2^ test for categorical variables. Pearson’s correlations were calculated between US FLI, A/G ratio, android percent fat, gynoid percent fat, triglycerides, cholesterol, HDL, LDL, HOMA-IR, and uric acid. Logistic regression was applied to assess the association between risk factors and NAFLD. Adjustments were made to the models. Model 1 was adjusted for age, race/ethnicity, marital status, and education levels. Model 2 included model 1 covariates plus BMI, hypertension, ALT, AST, gamma-glutamyl-transpeptidase, total cholesterol, triglycerides, HDL, LDL, uric acid, and glycated hemoglobin. We also conducted a logistic regression according to sex. A two-sided *p* < 0.05 was considered statistically significant. R 4.2.2^1^ was employed for all analyses.

## Results

### Baseline characteristics of the study population

A total of 10,989 participants (48.07% men and 51.93% women with a mean age of 44.37 ± 0.29 years) were included in this study, and 32.15% met the diagnostic criteria of NAFLD. The weighted baseline characteristics of the population are shown in Table 1. In contrast to individuals without NAFLD, those with NAFLD exhibited advanced age, higher values of body weight, BMI, waist circumference, glycohemoglobin, HOMA-IR, and uric acid, as well as worse lipid profiles. Additionally, they demonstrated an increased incidence of hypertension and diabetes, and a lower proportion of female participants. For regional body fat distribution investigated using DXA, android percent fat and the A/G ratio were notably higher in NAFLD participants than in NAFLD-free participants (41.68% vs. 32.80%, 1.14 vs. 0.94, *p* < 0.001, respectively), while gynoid percent fat showed a slight increase (37.23% vs. 35.24%, *p* = 0.003).

**Table 1:** Baseline demographics of NAFLD patients and NAFLD-free controls.

Variables	NAFLD-free controls	NAFLD patients	*p*
Age, year	37.91 ± 0.36	41.56 ± 0.51	<0.001
Female, %	52.37 (1.44)	44.08 (2.14)	<0.001
Race/ethnicity, %			<0.001
Non-Hispanic white	62.79(2.25)	59.91 (2.76)	
Non-Hispanic black	12.43 (1.27)	6.0 (0.97)	
Mexican American	7.66 (1.00)	17.34 (2.07)	
Other Hispanic	7.04 (0.97)	9.20 (1.27)	
Other race	10.09 (0.86)	7.0 (0.99)	
Marital status, %			
Married	52.25 (1.94)	56.19 (2.57)	0.001
Never married	25.54 (1.42)	21.23 (2.29)	
Living with partner	10.98 (0.85)	7.1 (1.11)	
Divorced	8.16 (0.79)	9.71 (1.55)	
Separated	2.19 (0.29)	3.20 (0.85)	
Widowed	0.88 (0.24)	1.96 (0.68)	
Education levels, %			<0.001
College Graduate or above	36.90 (2.02)	26.56 (2.31)	
Some College or AA degree	31.54 (1.58)	35.33 (2.38)	
High School Grad/GED or Equivalent	20.81 (1.47)	21.22 (1.72)	
9–11th Grade	8.14 (1.02)	11.07 (1.21)	
Less than 9th Grade	2.61 (0.45)	5.83 (0.89)	
Family PIR	2.99 ± 0.07	2.68 ± 0.08	0.021
Systolic blood pressure, mmHg	115.69 ± 0.37	123.35 ± 0.49	<0.001
Diastolic blood pressure, mmHg	69.28 ± 0.30	75.17 ± 0.44	<0.001
Hypertension, %	23.34 (0.95)	51.17 (2.22)	<0.001
Diabetes, %	5.93 (0.32)	28.60 (1.04)	<0.001
Height, cm	168.91 ± 0.26	169.59 ± 0.34	<0.001
Weight, kg	75.67 ± 0.41	100.44 ± 0.95	<0.001
Body mass index, kg/m^2^	26.46 ± 0.14	34.90 ± 0.31	<0.001
Waist circumference, cm	91.25 ± 0.33	113.50 ± 0.64	<0.001
ALT, U/L	21.27 ± 0.29	34.20 ± 0.89	<0.001
AST, U/L	22.73 ± 0.33	26.88 ± 0.65	<0.001
GGT, IU/L	19.10 ± 0.39	39.94 ± 1.62	<0.001
Total cholesterol, mg/dL	186.63 ± 0.99	196.25 ± 1.64	0.105
Triglycerides, mg/dL	101.12 ± 1.43	162.22 ± 3.85	<0.001
HDL, mg/dL	55.68 ± 0.46	45.10 ± 0.45	<0.001
LDL, mg/dL	111.00 ± 0.84	120.31 ± 1.39	0.146
Uric acid, mg/dL	5.11 ± 0.04	5.90 ± 0.06	<0.001
Glucose, mg/dL	92.12 ± 0.47	112.77 ± 1.62	<0.001
Glycohemoglobin, %	5.35 (0.02)	5.96 (0.05)	<0.001
HOMA-IR	1.92 ± 0.03	6.94 ± 0.31	<0.001
Total fat, kg	24.30 ± 0.27	37.85 ± 0.56	<0.001
Total lean, kg	49.48 ± 0.28	60.39 ± 0.58	<0.001
Android percent fat, %	32.80 (0.27)	41.68 (0.25)	<0.001
Gynoid percent fat, %	35.24 (0.25)	37.23(0.37)	0.003
Android to Gynoid ratio	0.94 ± 0.00	1.14 ± 0.01	<0.001
US FLI	11.35 ± 0.23	51.26 ± 0.83	<0.001

Data are presented as weighted mean ± SE or weighted percent (SE). ALT, alanine aminotransferase; AST, aspartate aminotransferase; BMI, body mass index; GGT, γ-glutamyl transpeptidase; HDL, high-density lipoprotein cholesterol; HOMA-IR, homeostasis model assessment of insulin resistance; LDL, low-density lipoprotein; NAFLD, nonalcoholic fatty liver disease; PIR, ratio of family income to poverty; US FLI, the United States fatty liver index.

### Association of the A/G ratio quartile with the prevalence of NAFLD

We applied the A/G ratio quartiles, the lower (0.90), median (1.00), and upper quartile (1.20), to divide the participants into four grades. The results showed that the prevalence of NAFLD was 5.60% in the first quartile and increased to 24.98%, 44.87%, and 58.32% in the second, third, and fourth quartiles, respectively (Table 2). The positive correlation between the A/G ratio quartiles and NAFLD prevalence suggests that individuals with a high A/G ratio are more strongly associated with NAFLD than those with a low A/G ratio.

**Table 2:** Association of the A/G ratio with the prevalence of NAFLD.

A/G ratio quartiles	PR%	PR	*F* value	*P*
1	5.60%	1.00		
2	24.98%	4.46		
3	44.87%	8.02		
4	58.32%	10.42	162.55	<0.001

A/G ratio, android to gynoid ratio; NAFLD, nonalcoholic fatty liver disease; PR, prevalence ratio; PR%, prevalence rate. A/G ratio quartiles: 1: <0.90; 2: 0.90–1.00; 3: 1.00–1.20; 4: >1.20. PR = the prevalence in test group (PT)/prevalence in control group (PC). The prevalence of PC was 5.60% when the A/G ratio quartiles were 1. The prevalence in A/G ratio quartiles over 1 was used as the PT.

### Correlation between fat distribution and other NAFLD risk factors

**Figure 1:**
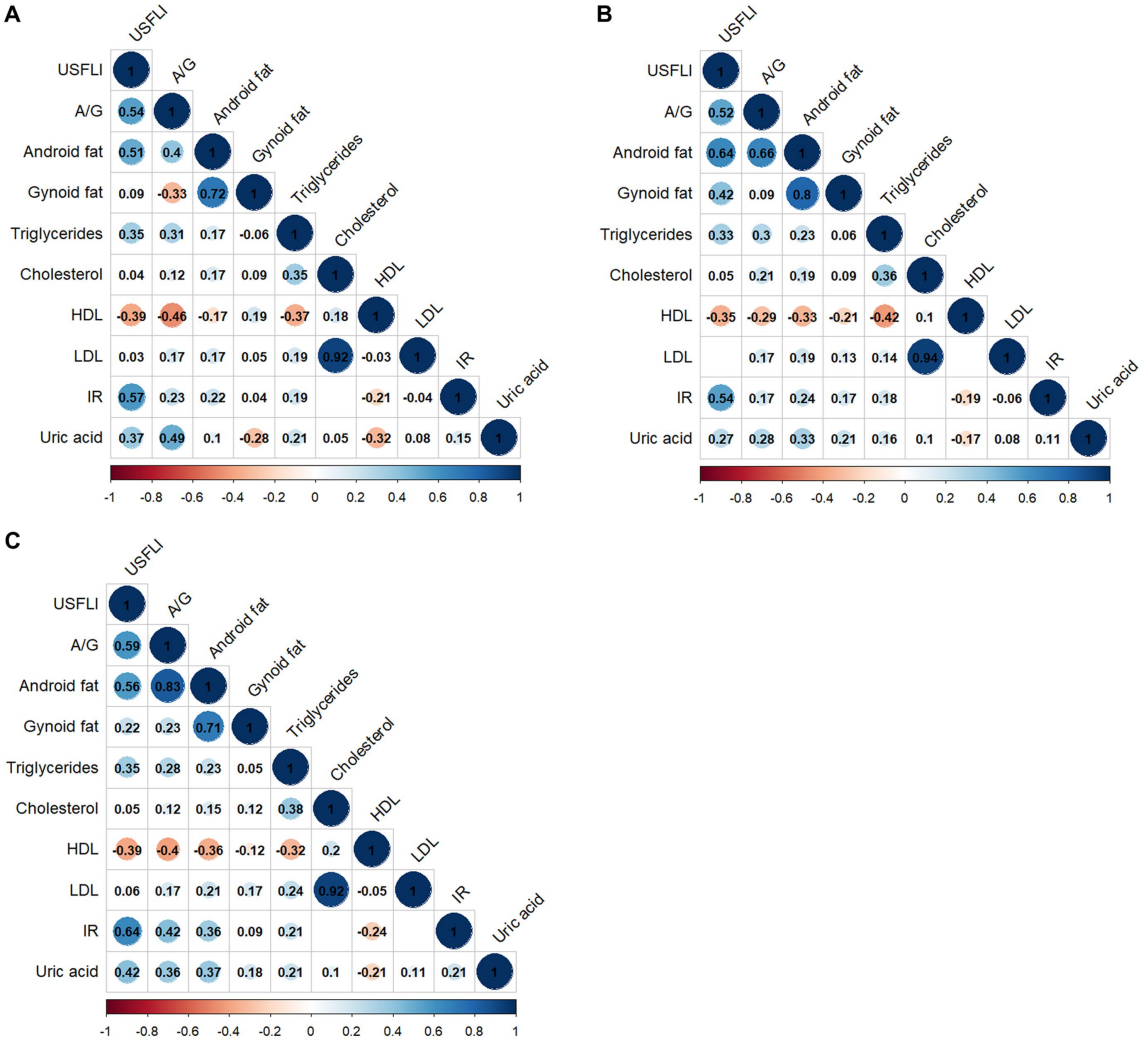
Correlation matrix of fat distribution and NAFLD-related risk factors by sex. **(A)** All people, **(B)** male subgroup, and **(C)** female subgroup. Only statistically significant Pearson’s correlations are shown in the matrix (*p* < 0.05). A/G, Android to Gynoid ratio; HDL, high-density lipoprotein cholesterol; IR, HOMA insulin resistance; LDL, low-density lipoprotein; US FLI, the United States fatty liver index.

A correlation matrix of adipose allocation and other NAFLD risk factors is summarized in Figures 1A–C for all individuals and for male and female groups, respectively. The A/G ratio was positively correlated with triglycerides, HOMA-IR, and uric acid but negatively correlated with HDL-cholesterol, as shown in Figure 1A. The correlation coefficient between the A/G ratio and triglyceride was 0.31 (*p* < 0.001). The correlation coefficient between the A/G ratio and HOMA-IR was 0.42 (*p* < 0.001) for females and 0.17 (*p* < 0.001) for males (Figures 1B,C). The A/G ratio and HDL-cholesterol had a stronger negative correlation in the female subgroup (r = −0.40, *p* < 0.001) than in the male subgroup (r = −0.29, *p* < 0.001). These findings suggested that the A/G ratio was correlated with dyslipidemia and insulin resistance and that there were sex differences.

### Logistic regression of fat distribution and prevalence of NAFLD

A complex sample logistic regression was used to investigate the relationship between fat depots and the prevalence of NAFLD (Table 3). In the crude model, android percent fat was positively related to NAFLD (OR: 1.36 95% CI: 1.34–1.39; *p* < 0.001) in the whole population, while gynoid percent fat was a negatively related to NAFLD (OR: 0.82, 95% CI: 0.81–0.84; *p* < 0.001). The A/G ratio proved to be a strongly related to NAFLD with an odds ratio of 3.45 (95% CI: 3.13–3.79; *p* < 0.001). Model 1 was adjusted for demographic factors, including age, race/ethnicity, marital status, and education levels. The odds ratios of the android, gynoid, and A/G ratio were 1.36 (95% CI: 1.34–1.39), 0.82 (95% CI: 0.81–0.84), and 3.36 (95% CI: 3.04–3.70), respectively. We further conducted multivariable logistic regression analyses, additionally adjusting for BMI, hypertension, diabetes, ALT, AST, gamma-glutamyl-transpeptidase, total cholesterol, triglycerides, HDL, LDL, and uric acid, in which there were similar OR values resembling the two previous models. The A/G ratio and android percent fat were still credibly related to NAFLD, and the gynoid percent fat was negatively related to NAFLD. Furthermore, we also found that a higher A/G ratio was significantly related to a higher prevalence of fibrotic NASH with an OR of 2.01(95% CI: 1.71–2.38, *p* < 0.001) after adjusting for age, race/ethnicity, marital status, education levels, BMI, hypertension, and diabetes (Table 4).

**Table 3:** Logistic analysis of fat distribution and NAFLD.

	Android percent fat		Gynoid percent fat		A/G ratio (SD)	
	OR (95%CI)	*P*	OR (95%CI)	*P*	OR (95%CI)	*P*
**Crude**						
Whole	1.36 (1.34–1.39)	<0.001	0.82 (0.81–0.84)	<0.001	3.45 (3.13–3.79)	<0.001
Male	1.35 (1.31–1.40)	<0.001	0.87 (0.84–0.9)	<0.001	3.70 (3.21–4.27)	<0.001
Female	1.34 (1.31–1.38)	<0.001	0.84 (0.8–0.87)	<0.001	9.62 (7.45–12.41)	<0.001
**Model 1**						
Whole	1.36 (1.34–1.39)	<0.001	0.82 (0.81–0.84)	<0.001	3.36 (3.04–3.70)	<0.001
Male	1.35 (1.30–1.39)	<0.001	0.88 (0.85–0.91)	<0.001	3.51 (3.01–4.09)	<0.001
Female	1.35 (1.31–1.39)	<0.001	0.83 (0.80–0.86)	<0.001	9.91 (7.54–13.03)	<0.001
**Model 2**						
Whole	1.15 (1.11–1.18)	<0.001	0.92 (0.90–0.94)	<0.001	1.59 (1.38–1.82)	<0.001
Male	1.11 (1.06–1.16)	<0.001	0.97 (0.92–1.01)	0.147	1.35 (1.10–1.66)	0.005
Female	1.14 (1.09–1.19)	<0.001	0.87 (0.83–0.91)	<0.001	3.01 (2.21–4.09)	<0.001

Model 1 was adjusted for age, race/ethnicity, marital status, and education levels. Model 2 was adjusted for covariates in Model 1 plus BMI, hypertension, diabetes, ALT, AST, gamma-glutamyl-transpeptidase, total cholesterol, triglycerides, HDL, LDL, uric acid. Android percent fat and gynoid percent fat were included in logistic regression at the same time in all models. The A/G ratio was analyzed alone without android percent fat or gynoid percent fat to avoid multicollinearity. A/G ratio, android to gynoid ratio; NAFLD, nonalcoholic fatty liver disease; OR, odds ratio.

**Table 4:** The association of the A/G ratio and fibrotic NASH.

	Fibrotic NASH	
	OR (95%CI)	*P*
**Crude**		
Whole	2.33(2.05–2.64)	<0.001
Male	1.91 (1.60–2.28)	<0.001
Female	3.87 (2.80–5.35)	<0.001
**Model 1**		
Whole	2.35 (2.04–2.70)	<0.001
Male	2.07 (1.69–2.53)	<0.001
Female	3.80 (2.64–5.48)	<0.001
**Model 2**		
Whole	2.01 (1.71–2.38)	<0.001
Male	1.63 (1.28–2.06)	<0.001
Female	2.33 (1.52–3.58)	<0.001

Model 1 was adjusted for age, race/ethnicity, marital status, and education level. Model 2 was adjusted for covariates in Model 1 plus BMI, hypertension, and diabetes. A/G ratio, android to gynoid ratio; NASH, nonalcoholic steatohepatitis; OR, odds ratio.

### Sex differences in the association between body fat distribution and NAFLD

Fat distribution and NAFLD categorized by gender are displayed in Table 5. More body fat in both the android area and gynoid areas was found in women than in men. In contrast, the A/G ratio was always lower in females, regardless of NAFLD status. These results showed that men and women have different fat distribution characteristics; despite more fat, women with lower A/G ratios distributed their fat more in the gluteofemoral area than in the abdomen.

**Table 5:** Features of body fat distribution by sex.

	General population			Non-NAFLD			NAFLD		
	Male	Female	*p*	Male	Female	*p*	Male	Female	*p*
Android fat mass (g)	2482.52 (32.70)	2512.13 (32.96)	0.426	1924.56 (23.44)	2072.20 (27.39)	<0.001	3649.47 (55.51)	4018.61 (64.03)	<0.001
Android percent fat (%)	32.18 (0.21)	38.73 (0.23)	<0.001	29.13 (0.21)	36.62 (0.24)	<0.001	38.56 (0.22)	45.97 (0.22)	<0.001
Gynoid fat mass (g)	4011.62 (39.54)	5412.12 (43.01)	<0.001	3536.84 (31.32)	5010.06 (40.42)	<0.001	5004.60 (75.34)	6788.93 (89.91)	<0.001
Gynoid percent fat (%)	28.56 (0.15)	42.56 (0.14)	<0.001	27.28 (0.16)	42.10 (0.16)	<0.001	31.25 (0.21)	44.14 (0.19)	<0.001
A/G ratio	1.12 (0.00)	0.91 (0.00)	<0.001	1.06 (0.01)	0.86 (0.00)	<0.001	1.25 (0.01)	1.05 (0.01)	<0.001

Data are presented as the mean (SE) or weighted percent (SE). NAFLD, nonalcoholic fatty liver disease.

**Figure 2:**
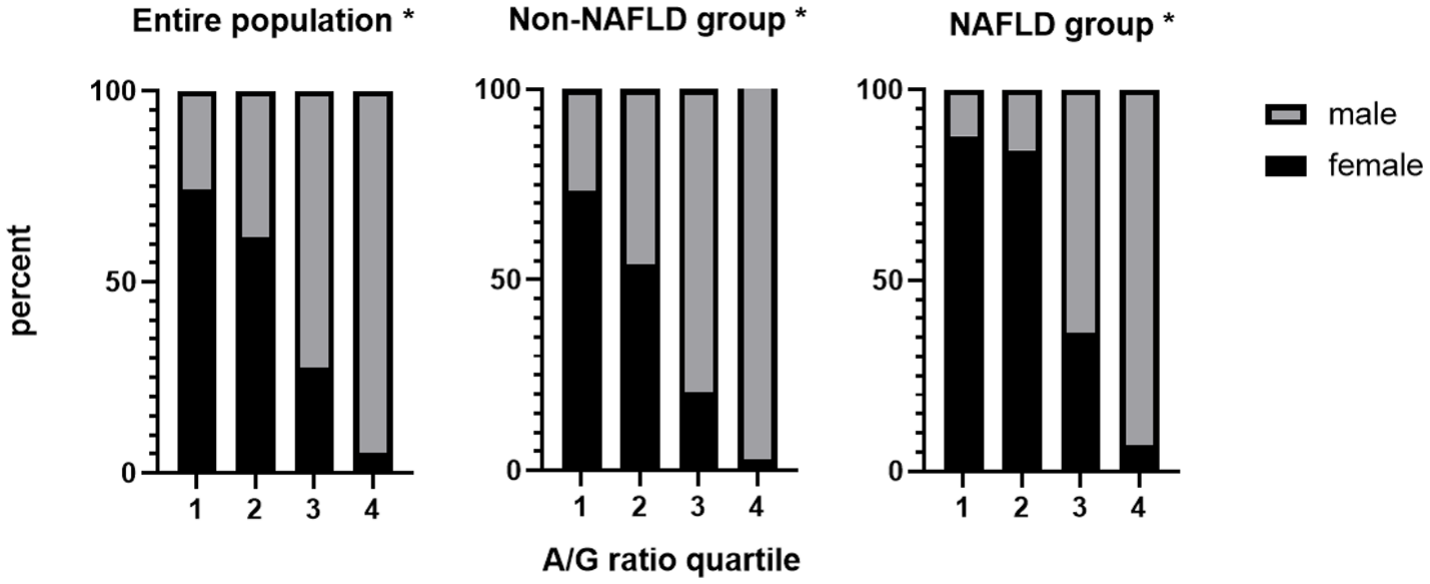
The female proportion decreased as the A/G ratio increased all groups. ^*^P for trend < 0.001. A/G ratio quartiles: 1: <0.90; 2: 0.90–1.00; 3: 1.00–1.20; 4: >1.20. A/G, android to gynoid ratio; NAFLD, nonalcoholic fatty liver disease.

We then divided the A/G ratio into quartiles and discovered a significant and strong negative correlation between the female proportion and the A/G ratio, particularly in the entire population and the NAFLD-free group. Overall, the NAFLD group showed a similar pattern, except for the first and second quartiles, in which the proportion of women did not decline correspondingly as in the other two groups (Figure 2). The result that female population proportion decreased as the A/G ratio grew, demonstrated that fewer females had high A/G ratios.

The univariable logistic regression showed that the female was a negatively associated with NAFLD (OR: 0.62, 95% CI: 0.55–0.69, *p* < 0.001). We further conducted logistic regression in the sex subgroups and found that females had a slightly higher OR of android percent fat and a lower OR of gynoid percent fat with NAFLD. For the A/G ratio, this association is more pronounced. The OR values of the A/G ratio in females were 9.62 (95% CI: 7.45–12.41), 9.91 (95% CI: 7.54–13.03), and 3.01 (95% CI: 2.21–4.09), respectively, which were critically higher than those in males in all three adjusted models [3.70 (95% CI: 3.21–4.27), 3.51 (95% CI: 3.01–4.09), and 1.35 (95% CI: 1.10–1.66), respectively] (Table 3). In addition, when analyzing the association of the A/G ratio and fibrotic NASH, we also found that females had higher OR values in all adjusted models (Table 4). These results implied that with the A/G ratio elevated, women were more related to NAFLD and fibrotic NASH than males.

### Sensitivity analyses of the association between the A/G ratio and NAFLD

In sensitivity analyses, we excluded extreme A/G ratio values [A/G ratio greater than 99% (1.5) or less than 1% (0.6)] and found that the association of the A/G ratio with NAFLD prevalence was not materially altered in all sex groups (Supplementary Table S1).

## Discussion

This cross-sectional study found a positive association between the A/G ratio and the prevalence of NAFLD, which fluctuated by sex. First, NAFLD participants had a notably elevated A/G ratio and android fat compared with non-NAFLD participants. Second, the A/G ratio was closely related to the prevalence of NAFLD. Third, the A/G ratio was correlated with dyslipidemia, insulin resistance and uric acid. Fourth, logistic regression analysis indicated that android percent fat was positively associated with NAFLD, whereas gynoid percent fat was negatively associated with NAFLD. In addition, the A/G ratio was found to be significantly associated with both NAFLD and fibrotic NASH, independent of BMI. Last, in the sex subgroup analysis, females had a lower A/G ratio than males, and the female proportion decreased as the A/G ratio increased. Females had a greater association of NAFLD and fibrotic NASH than males with a high A/G ratio.

In previous studies, obesity, defined mainly by weight or BMI (33), has been shown to be associated with the risk of metabolic diseases (34, 35). However, recent studies have found differences in the risk of cardiometabolic diseases and diabetes among individuals with a similar weight or BMI, potentially due to the different characteristics of fat distribution (36, 37). In this cross-sectional study, we provide new evidence that different regional fat depots have different threats independent of BMI: android percent fat in this study was proven to be positively related to NAFLD prevalence, whereas gynoid percent fat was negatively related to NAFLD. In addition, the A/G ratio, combining the two different fat depots for analysis, was significantly associated to NAFLD. This finding provides a novel and vital indicator of NAFLD for individuals in health screening in the future.

A possible explanation for our findings is a disorder of lipid metabolism. A study including a total of 627 Chinese women showed that android fat and the A/G ratio were significantly associated with higher odds of high triacylglycerols and low HDL-cholesterol, while gynoid fat was independently related to reduced odds (38). Individuals with high android fat and low gynoid fat tend to have excessive triacylglycerols, which might accumulate in hepatocytes in the long run and finally trigger the development of NAFLD (39). Another possibility is that different fat accumulation depots confer different susceptibilities to insulin resistance (40). A recent study highlighted that apple-shaped individuals (high android fat) had a higher risk of insulin resistance than BMI-matched pear-shaped (high gynoid fat) individuals (41). Aucouturier et al. also discovered a link between HOMA-IR and the A/G ratio (42). In this study, the A/G ratio was also found to be associated with a higher uric acid level, which may also explain the association between the A/G ratio and NAFLD. Uric acid has previously been shown to regulate hepatic steatosis and insulin resistance via the NOD-like receptor family pyrin domain containing 3 inflammasome and xanthine oxidase (43, 44).

It is a widely established fact that female adults have a lower epidemic of NAFLD, but there is no definite reason (3, 45). In this study, we found that females had a lower A/G ratio than males. Furthermore, the female population proportion decreased as the A/G ratio grew. This could partly explain the sex differences in the prevalence of NAFLD, as the A/G ratio is significantly associated with NAFLD. This study also noted that females with a higher A/G ratio are more strongly associated with the prevalence of NAFLD compared with males. In addition, morbid obesity was reported to be related to fibrosis of NAFLD by Ciardullo et al. (46). In this study, we also found that a higher A/G ratio was significantly related to fibrotic NASH, and this association is more apparent in females. What’ more, another study of 2,228 participants by Ciardullo et al. suggested that the A/G ratio was significantly related to liver fibrosis in NAFLD only in the female population and not in males (47). These findings indicated that females with a high A/G ratio were more dangerous not only for the occurrence of NAFLD but also for the progression of NAFLD. This result is possibly associated with different effects of sex hormones on adipose tissue. Sex steroid hormones were reported to have an direct effect on the metabolism, accumulation, and distribution of adiposity (48). Additionally, several loci displayed considerable sexual dimorphism in modulating fat distribution independent of overall adiposity (12, 49). This study identified a novel and significant association between the A/G ratio and NAFLD. In addition, women generally have a lower association with the prevalence of NAFLD than men; however, this advantage is reversed when the A/G ratio is higher. This indicated that the A/G ratio played a key role in the development of NAFLD in females. Investigating the mechanisms behind the link between the A/G ratio and NAFLD may provide a novel approach for the prevention and treatment of the disease.

Several limitations should also be acknowledged. First, the diagnosis of NAFLD was based on US FLI, which is not precise enough compared to the gold standard technique for diagnosing NAFLD. However, this score has been modified for the United States multiracial population and has a more accurate diagnostic capacity than the original FLI (22). To address racial disparities in the prevalence and severity of NAFLD, the US FLI includes race-ethnicity as a standard to enhance diagnostic capacity. When studying different populations, the race of the population should be fully considered in order to better diagnose NAFLD (50).

Second, US FLI is derived from a population aged 20 and older, so our study based on US FLI also used this standard, resulting in a lack of analysis of adolescents. Third, 40.3% (5,353/13,287) of initially qualified sample persons lacked DXA examinations. Given the lack of data, selection bias might exist. Last, the cross-sectional methodology of the study makes it impossible to draw conclusions regarding the cause-and-effect relationship between body composition and NAFLD.

## Conclusion

In summary, this cross-sectional study demonstrates that a higher A/G ratio is significantly correlated to higher NAFLD and fibrotic NASH prevalence. Given a higher A/G ratio, the weaker association between NAFLD and women is reversed. Additional studies investigating the reasons are needed.

## Data availability statement

The original contributions presented in the study are included in the article/Supplementary material, further inquiries can be directed to the corresponding author.

## Ethics statement

Ethical review and approval was not required for the study on human participants in accordance with the local legislation and institutional requirements. Written informed consent for participation was not required for this study in accordance with the national legislation and the institutional requirements.

## Author contributions

LY and CX conceived the study idea and designed the study. LY, HH, ZL, and JR performed the statistical analyses. LY wrote the manuscript. HH and CX revised the manuscript. All authors contributed to the article and approved the submitted version.

## Funding

This work was supported by the National Key Research and Development Program (2018YFA0109800), the National Natural Science Foundation of China (82070585), and the Key Research and Development Program of Zhejiang Province (2020C03033).

## Conflict of interest

The authors declare that the research was conducted in the absence of any commercial or financial relationships that could be construed as a potential conflict of interest.

## Publisher’s note

All claims expressed in this article are solely those of the authors and do not necessarily represent those of their affiliated organizations, or those of the publisher, the editors and the reviewers. Any product that may be evaluated in this article, or claim that may be made by its manufacturer, is not guaranteed or endorsed by the publisher.

## Footnotes

^1^ www.r-project.org/
