# Supplementary material for: Association of the android to gynoid fat ratio with nonalcoholic fatty liver disease: a cross-sectional study
Source: Front Nutr. 2023 May 15;10:1162079. doi: 10.3389/fnut.2023.1162079 (PMC10226647; doi:10.3389/fnut.2023.1162079)
Supplement: Supplementary file 1 [file Data_Sheet_1.docx]

**
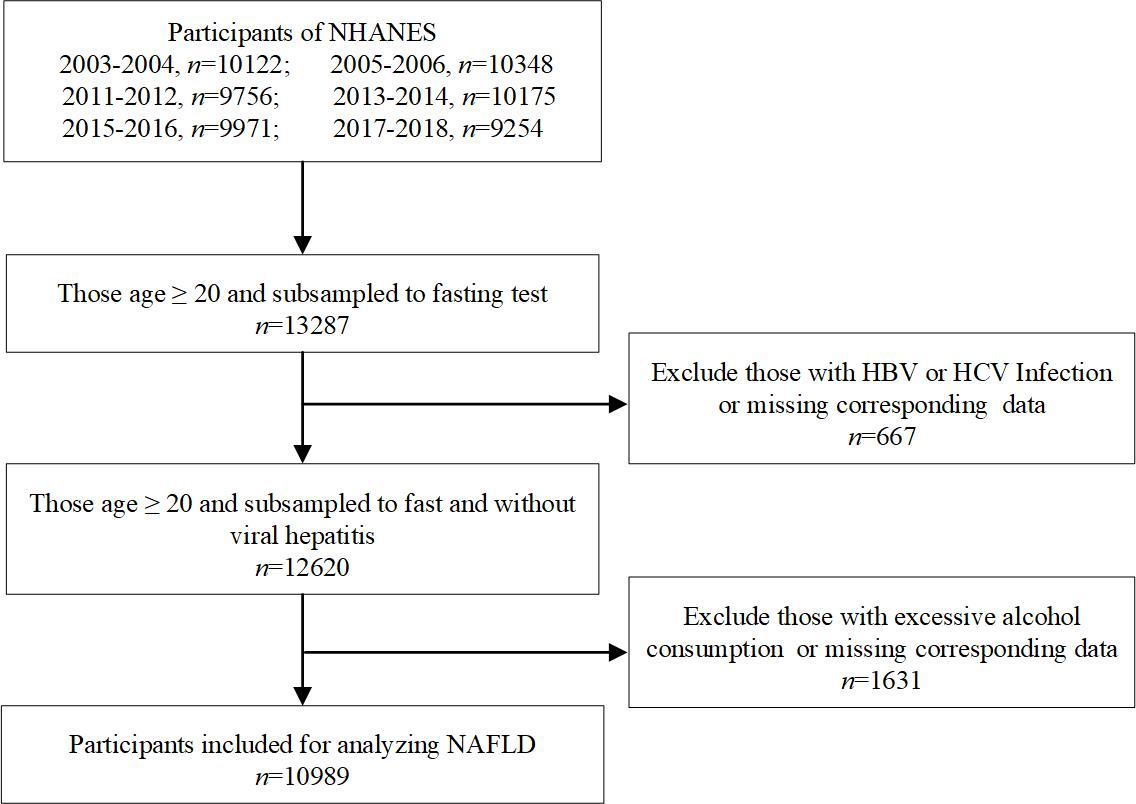
**

**Supplementary Figure S1.** Flow diagram of inclusion criteria from NHANES 2003–2006 and 2011–2018. HBV, hepatitis B virus; HCV, hepatitis C virus; NHANES, the National Health and Nutrition Examination Survey; NAFLD, nonalcoholic fatty liver disease.
