# Supplementary material for: Association of the android to gynoid fat ratio with nonalcoholic fatty liver disease: a cross-sectional study
Source: Front Nutr. 2023 May 15;10:1162079. doi: 10.3389/fnut.2023.1162079 (PMC10226647; doi:10.3389/fnut.2023.1162079)
Supplement: Supplementary file 2 [file Data_Sheet_2.docx]

**Supplementary Table S1.** Sensitivity analyses of the association of the A/G ratio with NAFLD

|  | **Exclude extreme A/G ratio^†^** | | |
| --- | --- | --- | --- |
|  | **OR** | **95%CI** | ***P*** |
| Whole | 1.60 | 1.38-1.87 | <0.001 |
| Male | 1.35 | 1.06-1.72 | 0.014 |
| Female | 3.01 | 2.21-4.09 | <0.001 |

Abbreviations: A/G, Android to Gynoid ratio; NAFLD, nonalcoholic fatty liver disease; OR, odds ratio, CI: confidence interval

**^†^** Participants with an A/G ratio greater than 99% (1.5) or less than 1% (0.6) were excluded.

The multivariate logistic regression model was adjusted for age, race/ethnicity, marital status, education levels, BMI, hypertension, diabetes，ALT, AST, gamma-glutamyl-transpeptidase, total cholesterol, triglycerides, HDL, LDL, and uric acid.

**Supplementary Table S2.** Correlation between waist circumference and the A/G ratio

|  | Pearson’s correlations | ***p*** |
| --- | --- | --- |
|  |  |  |
| waist | 0.601 | <0.001 |
| waist-to-height ratio | 0.485 | <0.001 |

Abbreviations: A/G, Android to Gynoid ratio;

**Supplementary Table S3.** The diagnostic ability of the A/G ratio in NAFLD

|  | **AUC (95%CI)** | **Cutoff** | **Sensitivity** | **Specificity** |
| --- | --- | --- | --- | --- |
| All participants | 0.771(0.752-0.789) | 1.05 | 0.725 | 0.689 |
| Males | 0.748(0.724-0.781) | 1.15 | 0.743 | 0.671 |
| Females | 0.821(0.800-0.849) | 0.95 | 0.812 | 0.680 |

Abbreviations: A/G ratio, android to gynoid ratio; NAFLD, nonalcoholic fatty liver disease; AUC, area under curve; CI: confidence interval

Note: The analysis was carried out among the sample participants.
